# From high to low malaria transmission in Zanzibar—challenges and opportunities to achieve elimination

**DOI:** 10.1186/s12916-018-1243-z

**Published:** 2019-01-22

**Authors:** A. Björkman, D. Shakely, A. S. Ali, U. Morris, H. Mkali, A. K. Abbas, A-W Al-Mafazy, K. A. Haji, J. Mcha, R. Omar, J. Cook, K. Elfving, M. Petzold, M. C. Sachs, B. Aydin-Schmidt, C. Drakeley, M. Msellem, A. Mårtensson

**Affiliations:** 1 Department of Microbiology, Tumor and Cell Biology, Karolinska Institutet, Solnavägen 9, SE-171 77 Stockholm, Sweden; 2 Health Metrics at Sahlgrenska Academy, University of Gothenburg, Gothenburg, Sweden; 3 Zanzibar Malaria Elimination Programme, Zanzibar, Tanzania; 4 MEASURE Evaluation, Dar es Salaam, Tanzania; 5 London School of Hygiene and Tropical Medicine, London, UK; 6 Department of Infectious Diseases, University of Gothenburg, Gothenburg, Sweden; 7 Centre for Applied Biostatistics, University of Gothenburg, Gothenburg, Sweden; 8 Biostatistics Unit, Institute of Environmental Medicine, Karolinska Institutet, Stockholm, Sweden; 9 Training and Research, Mnazi Mmoja Hospital, Zanzibar, Tanzania; 10 Department of Women’s and Children’s Health, International Maternal and Child Health, Uppsala University, Uppsala, Sweden

## Abstract

**Background:**

Substantial global progress in the control of malaria in recent years has led to increased commitment to its potential elimination. Whether this is possible in high transmission areas of sub-Saharan Africa remains unclear. Zanzibar represents a unique case study of such attempt, where modern tools and strategies for malaria treatment and vector control have been deployed since 2003.

**Methods:**

We have studied temporal trends of comprehensive malariometric indices in two districts with over 100,000 inhabitants each. The analyses included triangulation of data from annual community-based cross-sectional surveys, health management information systems, vital registry and entomological sentinel surveys.

**Results:**

The interventions, with sustained high-community uptake, were temporally associated with a major malaria decline, most pronounced between 2004 and 2007 and followed by a sustained state of low transmission. In 2015, the *Plasmodium falciparum* community prevalence of 0.43% (95% CI 0.23–0.73) by microscopy or rapid diagnostic test represented 96% reduction compared with that in 2003. The *P. falciparum* and *P. malariae* prevalence by PCR was 1.8% (95% CI 1.3–2.3), and the annual *P. falciparum* incidence was estimated to 8 infections including 2.8 clinical episodes per 1000 inhabitants. The total parasite load decreased over 1000-fold (99.9%) between 2003 and 2015. The incidence of symptomatic malaria at health facilities decreased by 94% with a trend towards relatively higher incidence in age groups > 5 years, a more pronounced seasonality and with reported travel history to/from Tanzania mainland as a higher risk factor. All-cause mortality among children < 5 years decreased by 72% between 2002 and 2007 mainly following the introduction of artemisinin-based combination therapies whereas the main reduction in malaria incidence followed upon the vector control interventions from 2006. Human biting rates decreased by 98% with a major shift towards outdoor biting by *Anopheles arabiensis.*

**Conclusions:**

Zanzibar provides new evidence of the feasibility of reaching uniquely significant and sustainable malaria reduction (pre-elimination) in a previously high endemic region in sub-Saharan Africa. The data highlight constraints of optimistic prognostic modelling studies. New challenges, mainly with outdoor transmission, a large asymptomatic parasite reservoir and imported infections, require novel tools and reoriented strategies to prevent a rebound effect and achieve elimination.

**Electronic supplementary material:**

The online version of this article (10.1186/s12916-018-1243-z) contains supplementary material, which is available to authorized users.

## Background

Substantial progress has been made globally in reducing the malaria burden during last decade [1] following the implementation and scaling up of vector control and effective treatment. This has led to increased international commitment to malaria elimination in several endemic areas [2, 3]. Elimination is considered feasible—and indeed has been achieved—using existing tools in areas of low to moderate transmission [4]. Whether elimination can be achieved in previously moderate to high transmission areas, however, remains unclear. According to the World Health Organization (WHO), “demonstrated technical feasibility in such eco-epidemiological settings is required [5].” Zanzibar with historically high malaria transmission [6, 7] may represent a rather unique, useful and valuable case study of such endeavour.

Zanzibar was among the first in sub-Saharan Africa to roll out wide-scale modern control interventions [8], i.e. the use of artemisinin-based combination therapy (ACT) in 2003/2004 followed by long-lasting insecticidal nets (LLINs), indoor residual spraying (IRS) and rapid diagnostic tests (RDTs) in 2005/2006 (Table 1).

**Table 1:** Implementation of malaria control tools/strategies in Zanzibar between 2002 and 2016

Year, month	Interventions
2002, November	New antimalarial treatment policy: ACT; 1st line: ASAQ, 2nd line: AL
2003, September	ACT deployment in all public health facilities.
2004	ITN distribution, geographically focused Intermittent preventive treatment in pregnancy (IPTp)
2005, September	LLIN universal distribution to all children < 5 years and pregnant women
2006, July	IRS (pyrethroid) aiming at annual universal coverage, in March before the main transmission season (after 2006)
2006	RDT provision to all public health facilities. LLIN provision initiated to all pregnant women and infants (9 months old) in MCH clinics
2008	LLIN universal distribution—two nets per household
2009	New antimalarial treatment policy: 1st line: ASAQ, 2nd line: quinine Weekly reporting of malaria cases by mobile phone from health care facilities (MEEDS)
2012	LLIN universal distribution—two nets per household IRS policy change: targeting hotspots only (carbamate 2012–2014, pirimiphos-methyl 2015-) Malaria case investigation and reactive household RDT screening and LLIN distribution
2015	RDT and ACT provision to private health facilities (AMFm programme) Intermittent screening and treatment in pregnancy (ISTp) replacing IPTp
2016	Larviciding in few selected sites New antimalarial treatment policy: ACT + primaquine (single low dose)

ACT artemisinin-based combination therapy, ASAQ artesunate-amodiaquine, AL arthemeter-lumefantrine, ITN insecticide-treated net, IRS indoor residual spraying, RDT rapid diagnostic test, LLIN long-lasting insecticidal net, MEEDS malaria early epidemic detection system, AMFm affordable medicines for malaria, MCH mother and child health

We herein report to what extent reduction in malaria transmission towards elimination has been achieved a decade after the intensified malaria control and why and where obstacles have emerged. Intervention uptake and temporal trends of a comprehensive variety of malariometric indices are presented including molecular and serological surveillance methods.

## Methods

### Study sites and malaria control interventions

**Fig. 1:**
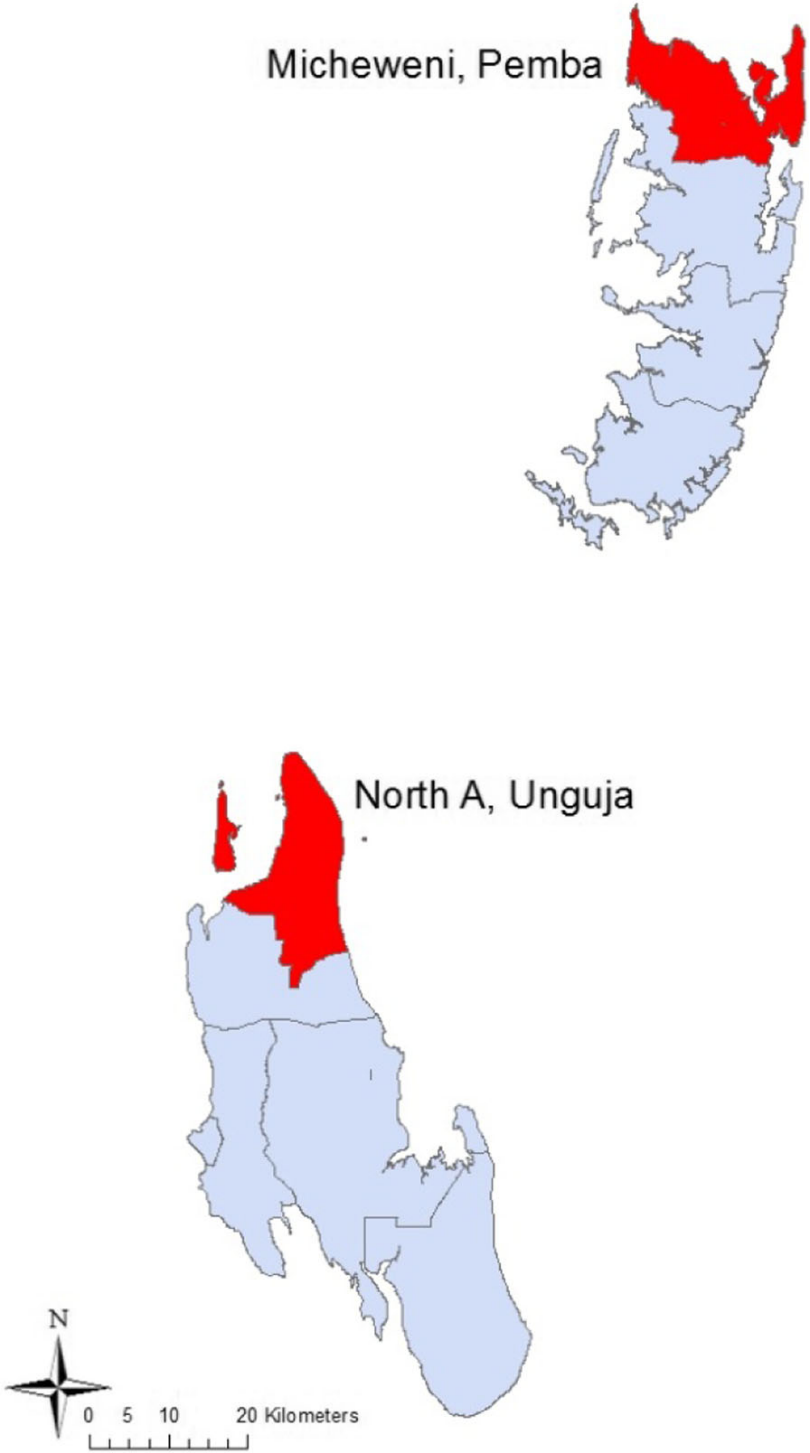
North A and Micheweni districts on Unguja and Pemba islands

Zanzibar (population 1.3 million) is an archipelago with two main islands about 30 km from mainland Tanzania (Fig. 1). The study was conducted in two rural districts, North A (Unguja island) and Micheweni (Pemba island), each with a population of approximately 100,000 people. The two districts, selected in 2003, were considered to be representative (e.g. mainly rural) for each of the two main islands of Zanzibar. Each of the two districts included one public district hospital, 13 public health care facilities and two private clinics. There is access to a public health care facility within 5 km distance throughout Zanzibar.

Malaria transmission has historically been holoendemic with a trend towards hyperendemicity in the 1990s [6, 7, 9] and perennial with two peaks associated with seasonal rainfalls in March–June and October–November. When the new interventions started in 2003, *Plasmodium falciparum* was the pre-dominant malaria species and *Anopheles gambiae* sensu *lato*, *An. funestus* and *An. coustani* were the main vectors [7].

The introduction of new malaria control interventions and strategies are presented in Table 1. The interventions were implemented by the Zanzibar Malaria Control Programme (ZMCP), which became the Zanzibar Malaria Elimination Programme (ZAMEP) in August 2013.

### Cross-sectional surveys

Nine cross-sectional household surveys were conducted in May–June between 2003 and 2015. The 2003 exploratory survey was conducted using a two-stage cluster sample technique. First, shehias (smallest administrative unit) and then the households were randomly selected from the sampling frame obtained from the Office of Government Statistics, Zanzibar [8]. This first survey included 625 households. Sample size calculations for the follow-up surveys were adjusted according to changing malaria prevalences. Those conducted in 2005 to 2007 were based on the proportion of children under five with malaria parasitaemia in 2003, 9%, and an assumed relative error of 20%. The calculated number of households to be included was 490 after adjusting for a design effect of 2. The same shehias were selected in the follow-up surveys, but each time with randomly selected households in proportion to the shehia size. From 2009 onwards, assuming 1% of households with at least one member being parasite positive and 95% CI of +/− 1% and 20% household members absent or refusing participation, the targeted sample sizes were 350 households.

Trained health personnel visited the selected households and conducted interviews (history of recent travel, access to health care, use of LLIN, IRS coverage, etc.) and blood sample collections (for malaria diagnosis by blood smear or RDT, PCR and serological analyses, etc.) upon written informed consent or proxy consent from legal guardians for children ≤ 15 years.

### Laboratory methods

Microscopic examination of Giemsa-stained thick blood smears was used for parasitological screening in the cross-sectional surveys 2003–2009. A *P. falciparum*-specific histidine-rich-protein 2 (HRP2)-based RDT (Paracheck) was used in 2011 followed by a combo RDT detecting both HRP2 and pan-*Plasmodium* lactate dehydrogenase in 2013 (SD-Bioline) and 2015 (Malaria ag combo).

*Plasmodium* detection by PCR was conducted on dried filter paper blood spots after DNA extraction by Chelex®-boiling method [10]. Cytb SYBR Green qPCR followed by restriction fragment length polymorphism analysis [11] was used for parasite screening and species determination. Parasite density was estimated by qPCR [12].

Antibody responses to three *P. falciparum* blood stage proteins (AMA-1, MSP-1 and GLURP) were determined by ELISA on blood spots from the cross-sectional surveys in 2009 and 2015 Mixture models were fitted to OD ELISA responses assuming two Gaussian distributions and the seroprevalence cut-off defined as the mean plus 3 standard deviations of the narrow, negative distribution [13]. Models were fitted separately for each antigen and combined to generate an overall seroprevalence if positive to one or more antigen. Seroconversion rates (SCRs) were estimated using reverse catalytic conversion models.

### Health facility and vital registry

Health facility data were collected monthly from the 26 public health care centres in the two districts (13 each) throughout the study period (1999–2015) using the Health Management Information System (HMIS) of the Ministry of Health. This included numbers of malaria-suspected patients, if malaria-tested and if confirmed by microscopy or RDT as well as individual characteristics of patients (age, sex, locality, etc.). In 2009, the HMIS data reporting was reinforced by the “malaria early epidemic detection system” (MEEDS) with weekly reports through a mobile phone reporting system (Table 1). Finally, from 2011, the “malaria case notification system” (MCN) was implemented, including added epidemiology-related information such as malaria risk factors (use of LLINs, IRS coverage, recent travel history, etc.) [14]. Parasitological confirmation by microscopy or RDT was not compulsory for all febrile patients until 2007. We therefore used positivity rates of those tested, rather than absolute malaria cases in our trend analyses.

Vital registry records on total births and deaths (1998–2014) were obtained from District Commissioner’s Office. Demographic data were obtained from the Tanzanian National Population and Housing Census.

### Entomological and meteorological data

Entomological data were collected from 22 sentinel sites (shehias) on Unguja and Pemba islands from 2005 onwards including North A and Micheweni districts. This included collections of adult mosquitoes indoors (18–06 h) and outdoors (18–24 h) in randomly selected households and larvae in selected breeding grounds. Indoor and outdoor human biting rates (HBRs) were determined by the human-landing catch method. In each sentinel site, two human volunteer baits would sit in- and outside two houses for four consecutive nights each month. Additional collections by pyrethrum spray light-traps and pit traps were also performed in some sites. Mosquitoes were stored in paper cups for species identification by PCR [15]. Sporozoites in the salivary glands were identified by ELISA [16] Insecticide resistance testing was conducted using the WHO guidelines [17]. *Anopheles* larva were collected, allowed to develop to adults, then exposed to the insecticides [15, 18].

Records of monthly rainfall for North A (1999–2015) and Micheweni (2005–2015) were obtained from Zanzibar Ministry of Communication and Transport through the Tanzania Meteorological Agency.

### Data management and analyses

Survey data were entered and validated using Microsoft Access and Excel. Recent surveys were conducted via tablets using Open Data kit. Statistical analyses were performed using STATA versions 12 and 13, and R version 3.2.2 (R Foundation for Statistical Computing, Vienna, Austria). Some data represent aggregate data from published articles [8, 15, 18, 19] and/or annual malaria reports of ZMCP/ZAMEP [14].

Pearson correlation coefficients were calculated to assess linear relationships between monthly rainfall and malaria incidence. Poisson regression model was used to assess malaria incidence and interaction of age (< 5 and > 5 years of age) and calendar year. Exact binomial tests were used where numbers were small (< 5). Otherwise, the normal approximation was used to test for differences in proportions. Trends in prevalences over time were tested using binomial logistic regression on the aggregate counts. Fisher’s test was used to assess differential reduction between malaria species. Logistic regression was used to assess the associations between risk factors and infection.

Serological conversion rate (SCR) was used to estimate the force of infection (incident malaria cases per population time) using maximum likelihood methods [20]. Temporal change in SCR was identified using profile likelihood plots and likelihood ratio tests against models with no change [3, 21].

Annual parasite incidence or index (API), defined as annual malaria cases per year per 1000 inhabitants, was based on any parasite-positive infection, whether symptomatic or not, whereas “clinical API” was based on reported malaria confirmed clinical patients only.

Reproductive control rate/ratio (Rc) is normally defined as number of new infections occurring from each index infection. We have estimated this concept as “annual Rc” in year X by calculating “annual incidence ratio” (incidence year X/incidence year X − 1) or “annual prevalence ratio” (prevalence year X/prevalence year X − 1).

The sporozoite rate was based on human light-trap catches throughout the year. However, after the intensified vector control in 2006, the sporozoite rates became too low for a precise estimate. The rate was then extrapolated from the mean human asexual parasite prevalence and density, assuming a linear correlation of the parasite prevalence and non-linear correlation of the parasite density [22] with the sporozoite rate (and infectiousness). The annual entomological inoculation rate (EIR) was determined as the mean HBR × mean sporozoite rate × 365 days.

## Results

### Intervention uptake

ACTs and RDTs were available in all health facilities without any documented stock-out periods since their respective implementation. There was high reported use of insecticide-treated net (ITN)/LLIN in all age groups in the cross-sectional surveys between 2006 and 2015 in Micheweni and North A (mean 68% and 74%, respectively) although children < 5 years were more likely to use ITN/LLIN (81%) than individuals ≥ 5 years (69%) (*p* < 0.0001) (Additional file 1: Figure S1). After the first IRS in July 2007, annual IRS was implemented in March each year prior to the expected main rains and transmission season. The mean proportion of households reporting having had IRS within the previous year was 87.6% from 2008 to 2011 in the two districts, 91% in Micheweni and 85% in North A—with annual ranges from 80 to 92% (Additional file 1: Figure S1). In 2013 and 2015 when IRS became more targeted, mainly to hotspot areas, the mean coverages were 79% in Micheweni and 54% in North A.

### Community-based parasite surveys

The overall microscopy- or RDT-determined parasite prevalences of all ages at the cross-sectional surveys are shown in Table 2 and Additional file 2: Table S1. Only *P. falciparum* infections were detected. In 2015, the prevalences were 0.6% in Micheweni and 0.3% in North A (mean 0.43%, 95% CI 0.23–0.73). This represents 95.9% and 96.6% (mean 95.8%; 24-fold) reductions in parasite prevalences, respectively, as compared with 2003. The main reduction occurred during 2006 and 2007 following the introduction of LLINs and IRS, after which a low prevalence was maintained, averaging 0.45% in Micheweni and 0.18% in North A. *P. falciparum* gametocyte carriage was detected at low prevalences (< 1%) in 2003–2006, but not in 2008–2009. The *P. falciparum* parasite densities in detected infections also declined significantly (Table 4). In the two districts combined, the geometrical mean parasitaemias were in 2003/51,147 parasites/μl (range 80 to 341,400) and in 2013/15,230 parasites/μl (range 53 to 770) among microscopy- and RDT (> 50 parasites/μl)-detectable infections.

**Table 2:** Community prevalences of asexual *P. falciparum* parasitaemia by microscopy or RDT; all age groups in May/June

Year	Micheweni district			North A district		
	Tested	Positive	Positivity rates (95% CI)	Tested	Positive	Positivity rates (95% CI)
2003	1189	172	14.5% (12.5–16.6)	2167	174	8.0% (6.9–9.3)
2005	1241	135	10.9% (9.2–2.7)	1503	48	3.2% (2.4–4.2)
2006	1182	56	4.7% (3.6–6.1)	1433	12	0.8% (0.4–1.5)
2007	1575	15	1.0% (0.5–1.6)	1499	0	0.0% (0.0–0.2)
2008	2091	10	0.5% (0.2–0.9)	1746	4	0.2% (0.1–0.6)
2009	1539	0	0.0% (0–0.2)	1163	0	0.0% (0.0–0.3)
2011*	1271	10	0.8% (0.4–1.4)	1561	2	0.1% (0.0–0.5)
2013*	1579	7	0.4% (0.2–0.9)	1447	3	0.2% (0.0–0.6)
2015*	1515	9	0.6% (0.3–1.1)	1497	4	0.3% (0.1–0.7)

*Malaria diagnosis by RDT instead of blood slide microscopy

The community parasite prevalences determined by PCR are presented in Table 3. Only *P. falciparum* and *P. malariae* were detected in the five surveys 2005–2015. The overall ratios between microscopy/RDT- and PCR-detectable malaria infections were 1:3 in 2005 and 1:8 in 2009–2015 combined. In 2015, the overall mean parasite prevalence in the two districts was 1.8% (95% CI 1.3–2.3%). This represents an estimated reduction of 92.7% (14-fold) compared with 2003. In the four surveys 2009–2015, the mean PCR determined parasite prevalence in Micheweni (3.2%) was 2.1 times higher than in North A (1.5%) (*p* < 0.001). However, there were opposite trends (2009–2015) in the prevalences, decreasing in Micheweni while increasing in North A (*p* < 0.001). There was no significant shift in relative prevalence from younger children to older age groups over time and throughout the study, the highest prevalences were seen in children 5–14 years. In 2005, the prevalences were 13.1% in children < 5 years, 36.4% in 5–14 years and 15.9% in > 14 years. In 2015, the prevalences were 1.9%, 2.0% and 1.7%, respectively.

**Table 3:** Community parasite prevalences (*P. falciparum* and *P. malariae*) by PCR; all age groups in May/June

Year	Micheweni district			North A district		
	Samples tested	Positive	Positivity rate (95% CI)	Samples tested	Positive	Positivity rate (95% CI)
2005	190	53	27.9% (21.7–34.9)	288	47	16.3% (12.2–21.1)
2009	1410	72	5.1% (4.0–6.4)	1013	9	0.9% (0.4–1.7)
2011	1378	45	3.3% (2.4–4.4)	1599	22	1.4% (0.9–2.1)
2013	1575	42	2.7% (1.9–3.6)	1448	26	1.8% (1.2–2.6)
2015	1519	26	1.7% (1.1–2.4)	1497	28	1.9% (1.2–2.6)

Linear trends in positivity rates from 2009 to 2015 were statistically significant in Micheweni district (decreasing *p* < 0.001) and in North A (increasing *p* = 0.036). These trends differed by district (interaction) (*p* < 0.001)

In the two districts combined, the main reduction of PCR-detected *P. falciparum* occurred from 2005 (20.9%) to 2009/11 (2.7%) followed by 2.0% in 2013–2015 whereas the main reduction of *P. malariae* occurred later, i.e. from 2005 (2.3%) and 2009–2011 (1.1%) to 2013–2015 (0.2%). Hence, the proportions of *P. malariae* infections among detected infections were 11/100 (11%) in 2005, 61/248 (25%) in 2009–2011 and 14/122 (11%) in 2013–2015 (*p* = 0.0004 for 2009–2011 vs. 2013–2015). The geometric mean parasite densities in 2009–2015 as determined by qPCR were 27 (range < 1–7825) parasites/μl among *P. falciparum* mono infections and 4 (range < 1–29) parasites/μl among *P. malariae* mono infections.

### Health facility data

**Fig. 2:**
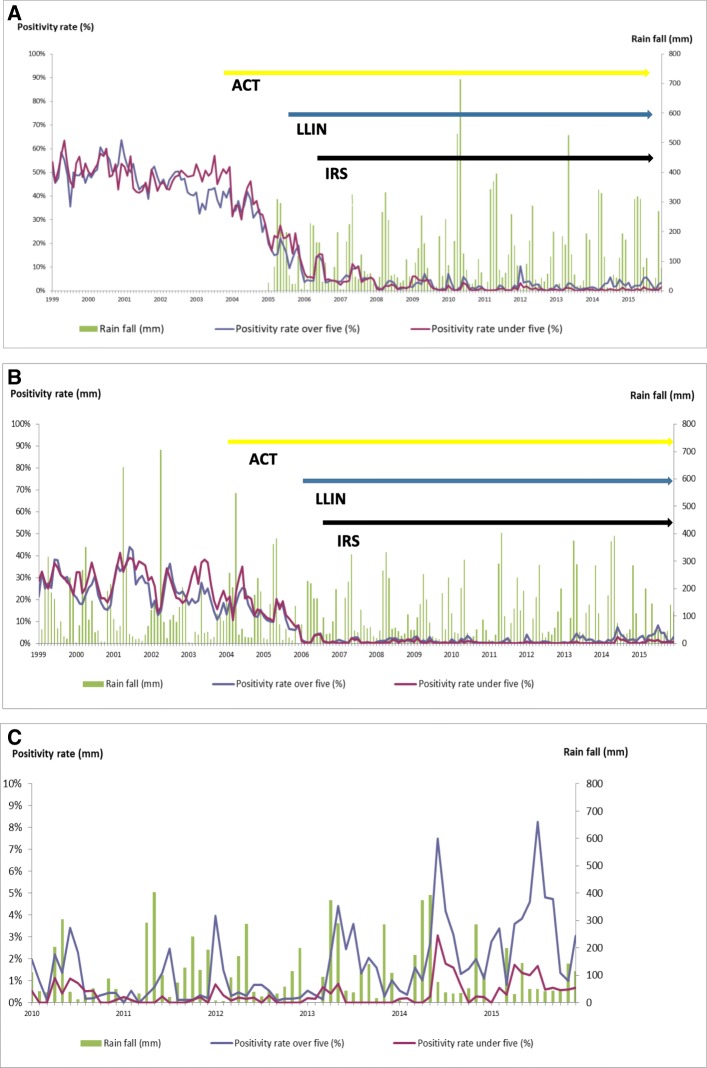
Malaria positivity rates among < 5 and ≥ 5 febrile patients in relation to monthly rainfall and interventions. **a** Febrile patients attending health care facilities in Micheweni district. **b**, **c** Febrile patients attending health care facilities in North A district

Monthly parasitologically confirmed malaria diagnoses from health facility outpatients reported between 1999 and 2015 are presented in Fig. 2a–c and Additional file 2: Table S2. The overall reduction in microscopy/RDT positivity rate between 2002 and 2015 was 95.7% (from 46.2 to 2.0%) in Micheweni and 89.7% (from 23.3 to 2.4%) in North A. This corresponds to 23- and 10-fold reductions in the respective districts and a mean reduction of 94.2% (Table 4).

**Table 4:** Malaria-related indices before interventions (2002–2003) compared to 2015; both districts combined

	2002/3	2015	Reduction % (X-fold)
Fever patients in health care facilities			
Parasite prevalence by microscopy/RDT	38.2% (95% CI 37.5–39.0)	2.2%, (95% CI 2.0–2.3)	94.2% (17)
Community-based cross-sectional surveys			
Parasite prevalence by microscopy/RDT	10.3% (95% CI 9.3–11.4)	0.43% (95% 0.23–0.73)	95.8% (24)
Parasite prevalence by PCR	24.8% (95% CI 23.4–26.3)	1.8% (95% CI 1.3–2.3)	92.7% (14)
Parasite densities			
Among microscopy positive or ≥ 50 par/μl—geometrical mean par/μl	1135 (Range 115–149,000)	161(Range 53–770)	85.8% (7)
Among all—arithmetical mean par/μl	450*	0.34	99.9% (1324)
Seroconversion rate per year	11% (95% CI 8–13)	0.8% (95% CI 0.6–1.1)	92.1% (14)
Crude under 5 child mortality per year**	1.01% (95% CI 0.84–1.19)	0.36% (95% CI 0.28–0.48)	64.4% (2.8)
Human biting rate per person night***	12.44*	0.27	97.8% (46)
Entomological inoculation rate (infective bites/year)***	136*	0.05	> 99.9% (2720)

*In 2005 before intensified vector control

**North A only

***Mean of indoors and outdoors

The main reduction occurred from 2005 when vector control was implemented in addition to ACT which is also consistent with the observed cross-sectional parasite prevalences. Between 2005 and 2008, the prevalence reduction was approximately 10-fold, i.e. with an annual reproductive control rate (Rc) of approximately 0.50, when estimated as “annual prevalence ratio”. The annual number of parasitologically confirmed malaria patients in the two districts went from 3528 in 2005, approximately sixfold down to 578 in 2008, with an annual Rc of approximately 0.55. From 2003 to 2008, the average annual Rcs were estimated to 0.50 and 0.63. From 2008 onwards, relatively low microscopy/RDT positivity rates were observed with averages of 1.8% in Micheweni and 1.2% in North A. The estimated average annual incidences (“Clinical APIs”) during this period were 3.6 and 2.0 (mean 2.8) per 1000 inhabitants for Micheweni and North A, respectively. Interestingly, there were opposite trends in the two districts from 2012 (*p* > 0.001) with continued decreasing parasite rates in Micheweni (*p* < 0.01) and increasing rates in North A (*p* < 0.01) (Additional file 2: Table S2).

A relative shift in parasite positivity rates was observed towards older age groups between 2002 and 2015 (Fig. 2a–c). Hence, the malaria patients < 5 years of age represented 47.2% (3067/6502) of all malaria patients in 2002 and 17.3% (125/721) in 2015 (*p* < 0.001) in the two districts. There was also a shift from perennial to seasonal malaria (during the main rains) (Fig. 2a–c). Hence, 2001 and 2002, 20% (374/1891) and 28% (380/1377) of reported malaria cases occurred during the 2 months with highest numbers whereas in 2014 and 2015 the corresponding figures were 44% (142/326) and 41% (128/316), respectively, i.e. a 1.8-fold increase comparing 2001/2002 to 2014/2015 (*p* < 0.001).

In Micheweni, prevalences of reported fever within 14 days prior to the cross-sectional surveys were 19% in 2003 and 7% in 2009 (*p* < 0.01) whereas in North A reported fever remained consistent around 12%. Both districts, reported high levels (> 90%) of public facility use, consistent with findings throughout Zanzibar from health management information systems in 2009 and 2010. The health facility reporting system lately included several private facilities and therefore most clinical malaria episodes in the study area.

### Risk factors for malaria infections

Risk factors associated with RDT-positivity among fever patients attending public health facilities, representing clinical malaria infections, compared to healthy controls, were assessed in 2015 (Table 5). History of recent travel (< 1 month) outside Zanzibar (mainly Tanzania mainland) was reported by 118 (44.2%) malaria-positive females and 244 (51.3%) malaria-positive males. Reported travel outside Zanzibar was associated with increased adjusted ORs of 85.6 (95% CI 50.9–151.4) for females and 60.3 (95% CI 38.9–96.5) for males, while travel within Zanzibar was not associated with any increased risk (adjusted OR, 95% CI 0.7–1.6). The temporal trends of the malaria cases reporting travel were similar to those not reporting travel, i.e. highly seasonal. Not sleeping under a bed net was also associated with increased risk of RDT positivity, adjusted OR 4.4 (95% CI 3.9–6.0), whereas IRS did not affect malaria risk.

**Table 5:** Risk factors in malaria infected asymptomatic and symptomatic individuals in both districts combined in 2015

	Healthy, malaria-negative controls****	Asymptomatic PCR positive	Unadjusted OR (95% CI)	Symptomatic RDT positive	Unadjusted OR (95% CI)	Adjusted OR (95% CI)
Total	2938 (100%)	53 (100%)	–	743 (100%)	–	1 (Ref)
Male	1202 (41%)	29 (55%)	1 (Ref)	476 (64%)	1 (Ref)	1 (Ref)
Female	1736 (59%)	24 (45%)	0.6 (0.3 to 1.0)	267 (36%)	0.4 (0.3 to 0.5)	0.5 (0.4 to 0.6)
Slept under net—yes*	2062 (70%)	33 (62%)	1 (Ref)	286 (38%)	1 (Ref)	1 (Ref)
Slept under net—no	876 (30%)	20 (38%)	1.4 (0.8 to 2.5)	457 (62%)	3.8 (3.2 to 4.5)	4.8 (3.9 to 6.0)
IRS—yes **	2059 (70%)	43 (81%)	1 (Ref)	492 (66%)	1 (Ref)	1 (Ref)
IRS—no	879 (30%)	10 (19%)	0.5 (0.3 to 1.0)	251 (34%)	1.2 (1.0 to 1.4)	1.2 (1.0 to 1.5)
No travel last month	2658 (90%)	50 (94%)	1 (Ref)	347 (47%)	1 (Ref)	1 (Ref)
Travel inside Zanzibar last month	233 (8%)	2 (4%)	0.5 (0.1 to 1.5)	34 (5%)	1.1 (0.8 to 1.6)	1.1 (0.7 to 1.6)
Travel outside Zanzibar last month	47 (2%)	1 (2%)	1.1 (0.1 to 5.3)	362 (49%)	59.0 (43.1 to 82.5)	70.2 (50.0 to 100.6)
No travel outside Zanzibar last year***	2811 (97%)	51 (98%)	1 (Ref)	No data	–	–
Travel outside Zanzibar last year	80 (3%)	1 (2%)	0.7 (0.1 to 5.1)	No data	–	–

*Slept last night before survey under a net

**IRS within last year before survey

***Travel from 1 month to 1 year before survey

****Asymptomatic PCR negative individuals in cross-sectional survey

Risk factors associated with asymptomatic PCR positivity were also assessed in the 2015 cross-sectional survey (Table 5). There was no evidence to suggest that malaria infection was significantly associated with travel outside or inside Zanzibar (past month or within last year) or with use of any vector control.

### All-cause child mortality

**Fig. 3:**
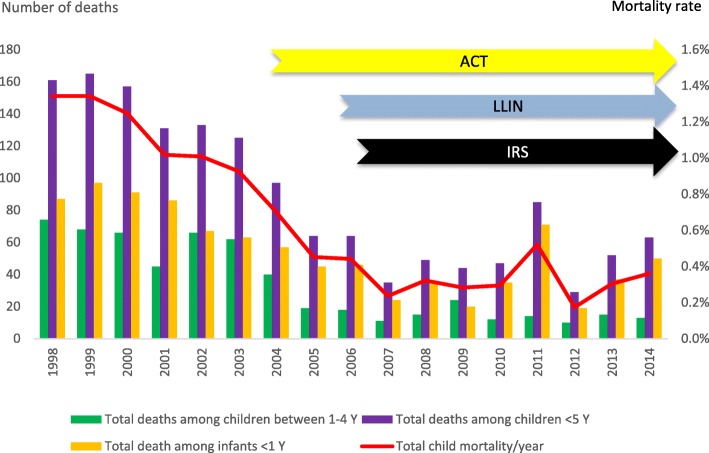
All-cause mortality in children < 5 years of age in North A district

The Vital Registry reported all cause annual mortality rate among children < 5 years in North A district was 1.01% in 2001/2002 before the malaria control interventions (Additional file 2: Table S3). The mortality rate then decreased to 0.45% in 2005 after introduction of ACTs in September 2003 and was further reduced to an annual mean of 0.28% in 2007/2008 after introduction of ITNs and IRS (Fig. 3). In 2009–2014, the mean rate was 0.32. This corresponds to a 72% (95% CI 6579) reduction between 2002 and 2007 and estimated under 5 mortalities of 51 (2001/2) and 14 (2007/8) per 1000 births. The observed decrease was most pronounced in children aged 1–4 years with an estimated 79% reduction from an annual mortality rate of 0.53 to 0.11% in 2001/2002 and 2007/2008 respectively. For children < 1 year, the corresponding reduction was 67% from 2.93 to 0.96% annual mortality rates.

### Seroconversion and force of infection

**Fig. 4:**
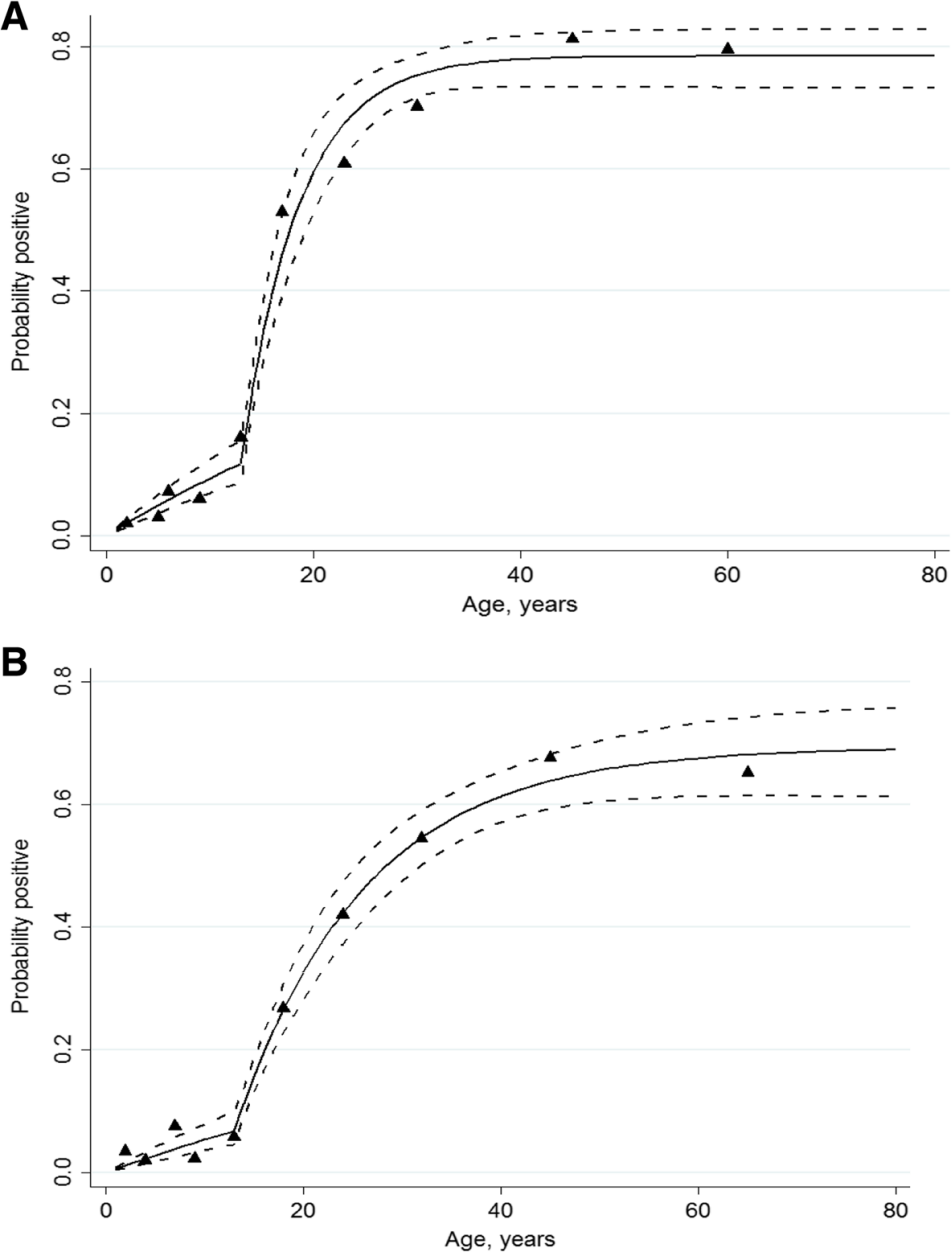
Age-related prevalences of anti-*P. falciparum* antibodies in Micheweni district (**a**) and North A district (**b**)

Seroprevalences to *P. falciparum* antigens increased with age in both settings (Fig. 4a, b). Profile likelihood analysis identified that models with two SCRs fitted better than a single force of infection with a change at approximately 5 years of age in the 2009 data and approximately 13 years of age in the 2015 data, consistent with the scale up of interventions between 2003 and 2006 (Fig. 4a, b). Estimates of force of infection in 2015 suggest that current SCRs are 10 and 16 fold (90%, 94%) lower than before interventions: North A previous SCR 0.06 year^− 1^ (95% CI 0.04–0.09), current SCR 0.006 year^− 1^ (0.004–0.008), and Micheweni previous SCR 0.16 year.^− 1^ (0.11–0.23), current SCR 0.010 year.^− 1^ (0.007–0.014). The average current SCR of 0.008 year.^− 1^ (0.006–0.011) would correspond to an estimated annual parasite incidence (API) of about 8 cases (between 5 and 20) per 1000 inhabitants.

### Entomological findings

In 2005, before the intensified vector control interventions among 2203 *Anopheline* mosquitoes collected in the sentinel sites of Unguja and Pemba, 2187 (99%) were *An. gambiae* s.l., 11 *An funestus* and 5 *An coustani*. In 2010/2011 among 2837 collected anophelines in Micheweni and North A, 2702 (95%) were *An. gambiae s.l.* and 94 (3%) *An. funestus*. Other species included *An. rivulorum* (*n* = 19), *An. hancocki* (*n* = 8), *An. zeimani* (*n* = 8) and *An. maculipalpis* (*n* = 6). Among *An. gambiae s.l.,* there has been a shift in the longitudinal sentinel sites from relatively few *An. arabiensis* in 2005 (6/160; 4%) to becoming predominant 2007–2014 (2907/3191; 91%) supporting the increasing relative importance of outdoor biting/resting mosquitoes for malaria transmission (Additional file 1: Figure S2). *An. merus* has been the second most frequent subspecies, with proportion ranging from 1 to 7% in different collections and years. Larvae collections in 2012/2013 provided similar findings with 1190/1382 (86%) *An. arabiensis* and 113/1382 (8%) *An. merus*.

In 2005, the mean *Anopheline* HBRs (in- and outdoor combined) were 16.30 and 8.57 (mean 12.44) bites/man night during January to June in Pemba and Unguja, respectively, before the first LLIN distribution. With an average sporozoite rate of 3.0% (36/1194), the estimated annual EIRs were 178 and 94 (mean 136) infected bites respectively, with large fluctuations during the year. In 2015, the mean HBRs were 0.39 and 0.14 (mean 0.26) on the two islands (Table 4). The indoor HBRs were 0.02 and 0.13 (mean 0.08) bites/man night whereas the outdoor HBRs were 0.75 and 0.14 (mean 0.45). Assuming a correlation between sporozoite rates and asexual parasite rates and densities [22], the sporozoite rate in 2015 may be possibly estimated to 60 (20 × 3) times lower than in 2005, i.e. a mean of 0.05%. The annual EIRs in 2015 would then be 0.07 and 0.03 (mean 0.05) on Pemba and Unguja islands respectively.

The average reduction in overall HBR between 2005 and 2015 maybe estimated to 97.8% (Table 4). The estimated mean annual EIRs then decreased from 136 to 0.05 infected bites (Table 4), i.e. over 2000-fold (> 99.9%). In 2015, most bites (65%) occurred during three peak months (November and April/May) compared to 52% during the same months in 2005.

Pyrethroid resistance was first detected in 2010 in Pemba [18] and has now spread throughout Zanzibar [19]. Hence, resistance tests performed 2012–2014 with different pyrethroids resulted in 24 to 88% and 33 to 82% 24 h (mortality of the main vectors in Unguja and Pemba, respectively ([15], and new data). In contrast, bendiocarb and pirimiphos-methyl (Actellic 300CS, Syngenta) were still 100% effective against the malaria vectors in 2014 [18].

### Rainfall and malaria

The annual rainfall was rather consistent in North A between 1999 and 2015, with the exception of 2003, 2010 and 2015 (Fig. 2a–c). The mean annual rainfalls for these 3 years were 48%, 28% and 36% lower than the mean annual rainfall (1345 mm). A significant correlation was observed between monthly rainfalls and confirmed malaria diagnoses from 2007 to 2015 (Pearson correlation coefficient [rp] = 0.37, *p* < 0.01), but not in the previous period of monitoring (1999–2006) ([rp] =0.04, *p* = 0.78 and [rp] = 0.33, *p = 0*.*11*).

## Discussion

This study provides an explicitly detailed description of the decline in malaria transmission in two districts of Zanzibar over a 12-year period. Reductions of more than 90% were observed in prevalence of infection, incidence of malaria and human biting rate. A major drop in child mortality was seen already following the introduction of ACTs in 2003, whereas the major drop in malaria transmission occurred after the introduction of vector control in 2005.

### Coverage and sustainability of malaria control interventions

An overall good access to public health care, a continuous supply and adherence to RDTs and ACTs [23–25] as well as sustained ACT efficacy [26, 27] have supported a continued efficient management of clinical malaria episodes in all age groups.

Quite high and sustained coverage of effective vector control has been achieved, generally higher than in other sub-Saharan countries according to their national reports [1]. Iterated household level distributions of LLINs with health information have resulted in consistently high degrees of reported use in children < 5 years and other age groups. Combined with high coverage of IRS, this appears to have had a significant (almost 100-fold) effect on the indoor vectorial capacity, as may be forecasted [28, 29]. Both interventions are publically perceived as beneficial against malaria and biting insects in general [30, 31]. However, the recent increase in pyrethroid resistance represents a major concern [19]. The more costly carbamate or pirimiphos-methyl has therefore replaced pyrethroids in IRS whereas the pyrethroid impregnated LLINs may still provide relative protection [32, 33]. Additionally, the change in biting behaviour of malaria vectors suggests outdoor malaria transmission poses another challenge to malaria elimination in Zanzibar. A similar shift in species proportions has been reported in other areas of Africa following wide-scale LLINs use and IRS programmes [34–37]. Complementary antivectorial efforts targeting both pyrethroid resistant and outdoor resting mosquitoes are clearly required for successful malaria elimination in Zanzibar.

### Malaria transmission impact

The initial rapid reduction in transmission from 2005 to 2007 in the two study districts [8] (annual Rc about 0.5) was followed by a much slower decline with persistent low-level transmission from 2008 onwards. Although we focussed on two districts only, they appeared to be representative for all of Zanzibar. The malaria positivity rates in febrile patients reported from the health facilities in the other eight districts were quite similar to the rates in the two study districts during the study period [14]. A new equilibrium of malaria pre-elimination stage transmission thus appears established despite continuing high community uptake of the conventional control interventions.

The reductions in malaria indices in Zanzibar are much more pronounced (around 5- to 10-fold higher) than those reported from other areas/countries of sub-Saharan Africa including Tanzania mainland [1, 38–43]. In addition, these gains appear to be more sustained in Zanzibar than in many other areas where a tendency of resurgence may be occurring in the latest years [1]. This highlights the uniqueness of the malaria elimination efforts in Zanzibar. There are probably multiple reasons for the effectiveness but we believe a major factor is higher population-level uptake of the interventions due in large part to the strong commitment of ZAMEP and the Zanzibar Government and strong involvement of the communities. An easy access to health care and accurate malaria treatment is probably also essential. We do not believe other general factors such as socioeconomic changes and sudden improvement in health care may have strongly influenced the impact on the rapid 10- to 100-fold reduction in malaria indices. A socio-economic development has probably occurred in Zanzibar as in many other areas of Africa during the study period, but would only account for a minor part of the malaria control impact.

The establishment of this new persistent level of low transmission contrasts with the malaria elimination feasibility report for Zanzibar based on mathematical modelling which predicted a possible annually continuous Rc of 0.5 and elimination achieved by 2020 if effective intervention coverage was kept at approximately 75% [44]. There may be several reasons why this prediction became unrealistic. Firstly, malaria transmission is now more seasonal and geographically heterogeneous with clear foci of infection [14], which may be driving current transmission [45]. Secondly, sensitive molecular testing by PCR has highlighted the major reservoir of low-density asymptomatic parasitaemias across all age groups, which appears consistent with other low transmission areas [46–48]. Importantly, these low-density parasitaemias may contribute significantly to the residual ongoing transmission [49, 50] and interestingly this appears to be possible despite a major decline in HBRs (Table 4). An additional interesting finding was that the relative proportion of low-density *P. malariae* infections increased after the initiation of interventions up to 2011. This may reflect greater longevity of untreated asymptomatic *P. malariae* than *P. falciparum* infections [51] but relatively lower presently ongoing transmission.

Thirdly, imported malaria may represent a major hindrance for elimination. A relative risk factor for clinical malaria infection was indeed history of travel outside Zanzibar. Such travel, mainly to/from Tanzania mainland, was reported by 49% of clinical malaria patients (OR 70) in 2015. Neither recent nor previous travel were significant risk factors for asymptomatic infections. In 2010, travel outside Zanzibar was reported by only 9/121 (7%) malaria-confirmed patients (OR 9) [23]. In 2013, travel outside of Zanzibar was reported by 30% of reported clinical malaria patients [14]. A tentative interpretation of this may be that an increasingly important fraction of new clinical infections are acquired from outside Zanzibar although still somewhat less than reported estimates for Zanzibar based on modelling [52]. Conversely our cross-sectional data, including the serology results, may suggest that many infections are locally acquired and possibly asymptomatic and thus remain as untreated residual infections for a significant period of time allowing for maintained residual local transmission [53]. Hence, the official figures in Zanzibar of approximately 3000 malaria confirmed clinical infections significantly underestimate the actual incidence of newly acquired infections. Such figures may be better reflected by SCR estimates. Extrapolating the mean SCR for the two study districts (0.008 year^−1^) to whole of Zanzibar would suggest over 10,000 new infections annually.

A fourth reason for the halt in malaria transmission reduction despite the significant reduction of HBRs is probably that the residual vector population mainly biting and resting outdoors is now less affected by indoor vector control. Hence, not having IRS recently performed was not identified as a major risk factor, while not sleeping under LLIN was only a risk factor for clinical malaria, again supporting that most transmission may occur outdoors. It also suggests that the preventive mass effects of LLIN use and IRS on indoor transmission are now more significant than the individually preventive effects. However, the spreading resistance to pyrethroids [19] and more restricted coverage of IRS represent challenges for future sustained impact, although the exact epidemiological effects of resistance to the insecticide in the nets appear to vary [32, 33], and indoor HBRs presently remain very low in our study area.

A fifth potential challenge is that malaria immunity is expected to decline as transmission reduces [54]. An increased proportion of clinical malaria episodes was seen among patients > 5 years although such age shift was not yet seen among PCR detected low-density parasitaemias. These older age groups were previously likely to be clinically immuno-protected through previous repeated exposure. A reason for the relative age shift may however also be behavioural. Older age groups who remain outside in the evening are exposed to more outdoor biting mosquitoes and may be less prone to use LLINs against mosquitoes biting indoor. Finally, it may also result from more frequent travel to mainland Tanzania by older age groups.

It is commonly stated that it is easier to control/eliminate malaria on an island (ex Zanzibar) than in-country (e.g. Tanzania mainland). This is may be true for a small island with a small population [55]. However, we do not believe this to be a major reason for the high impact on the two large islands of Zanzibar with populations over half a million each. There is obviously increased risk of imported malaria on the African continent between neighbouring countries through more crossing of borders as well as to some extent exchange of mosquitoes to nearby areas. But grossly besides the very border areas there should not be major differences between the malaria control efforts required in Zanzibar islands and mainland Tanzania. We therefore consider the findings and challenges in Zanzibar highly applicable to other African countries.

### Public health impact

The study data provide evidence of a major improvement in child health. A highly significant reduction of all cause child mortality coincided with the introduction of ACT whereas the most significant reduction in malaria incidence occurred after the intensified vector control. Although there was a trend of reduction in under 5 child mortality already before 2003, the decline 2003–2005 was clearly more pronounced.

The reported child deaths to the Vital Registry probably represent an underestimated mortality but we believe the reporting rate remained rather similar during years of study, allowing for reasonably valid trend analysis. In addition, the specifically large mortality reduction during 2003–2006 cannot be explained by any other public health intervention at that time in Zanzibar. A major decrease in crude child mortality was also observed on Bioko Island after massive malaria control interventions [42]. In parallel to our observed mortality reduction, there was also reduced hospitalisation for severe malaria [56] and major reduction in severe anaemia requiring blood transfusions to < 5 children [8], a common severe manifestation of malaria in sub-Saharan Africa. The overall reduction of clinical malaria episodes resulted in a decline in fever episodes especially in children, also noted by their caretakers and thus a good incentive for sustained LLIN use [30, 31].

The strong impact on crude mortality may largely be explained directly by reduced malaria specific mortality from ACT preventing development of severe malaria manifestations and the vector control reducing malaria incidence. It may however also result indirectly from the general reduction of malaria infection and its associated anaemia, being a risk factor for severe manifestations of other concomitant bacterial infections [57], e.g. septicaemia [58] or pneumonia.

### Additional tools and strategies required

A new low malaria transmission epidemiology has emerged in Zanzibar with spatial, temporal and demographic foci of infection. These foci are likely to be influenced by outdoor transmission and increasing insecticidal resistance, a substantial asymptomatic parasite reservoir and an apparent increasing number of imported infections. All this necessitates the addition of new malaria control tools and strategies.

Screening (by RDT) and subsequent preventive treatment of asymptomatic but possible parasite carriers has now been introduced in households where clinical malaria episodes have been identified and potentially in the future within for example 300-m radius [59]. Since 2012, IRS with carbamate and pirimiphos-methyl has been specifically targeting identified hotspots and larvicing interventions are being trialled in selected foci. Gametocytocidal single low-dose primaquine is being introduced along with ACT. General surveillance is also being reinforced by more comprehensive and regular epidemiological investigations of newly detected and reported cases, as well as more comprehensive monitoring of entomological insecticide resistance and parasitological drug resistance.

However, other possibly more aggressive approaches are also needed. This may include screen and treat strategies potentially including new highly sensitive diagnostics [60–62] or targeted mass/focal drug administration [59, 63] possibly including seasonal chemoprevention [64, 65]. These actions may be targeted to hotspot areas [66] and/or population groups at risk of residual parasite reservoir. Additional vector control targeting *An. arabiensis* populations needs to be implemented, e.g. different outdoor mosquito “attract and kill” methods. Case detection and response also needs further development to ensure future rapid prevention of outbreaks especially from imported infections. Since imported malaria represents a significant barrier to malaria control and elimination efforts in Zanzibar, several additional preventive strategies may be considered, especially during high transmission season. Such interventions may include chemoprophylaxis to Zanzibar is travelling to mainland, and mass screening or presumptive treatment of anyone arriving from mainland. However, formal and informal exit and/or entry points from/to Zanzibar are numerous and the efficiency of for example screening and treatment may not be very significant with standard diagnostic tools [61]. This will be explored in Zanzibar in the near future.

With old reinforced and new introduced interventions, the Rc presently around 1 may possibly be reduced to 0.5 again. The presently annually reported 3000 clinical malaria cases and our estimated 10,000 infections would then be reduced below 10 and a state of elimination potentially achieved by approximately 2026.

## Conclusions

Zanzibar has made huge progress towards malaria elimination, differently from most other areas in sub-Saharan Africa where recent progress appears difficult to sustain. However, bending the transmission curve further requires significant thought, impetus and funding. In the past, there have been attempts to strongly control/eliminate malaria in Zanzibar although not as successfully as now, but malaria has resurged each time due to high vulnerability and receptivity. Zanzibar is therefore now embarking on a new roadmap. The data presented and the obstacles and challenges identified in the present study suggest that responding to these challenges with reoriented and new strategies Zanzibar may possibly accelerate the reduction and elimination of local malaria transmission within a coming 10-year period. This may then provide a highly wishful proof of elimination concept from a high endemic area. However, clearly, the global community presently expresses overoptimistic ambitions regarding the timing of malaria elimination, relying on views and modelling which grossly underestimate the challenges ahead related to both escaping mechanisms of the parasite and mosquito as well as operational constraints.

## Additional files

Additional file 1: **Figure S1.** Proportions of < 5 and ≥ 5 years of age reporting having slept under a bed net the night before and proportion of households reporting having been sprayed the year before the respective surveys between 2003 to 2015 in a) Micheweni district and b) North A district. Figure S2 Proportions of *An. arabiensis* among *An. gambiae* s*.l* mosquito samples collected in surveys on Unguja island, Zanzibar between 2005 and 2014. (DOCX 100 kb)

Additional file 2: **Table S1.** Community prevalences of *P. falciparum* asexual parasitaemia and gametocytaemia according to microscopy of RDT by age group in North A and Micheweni districts of Zanzibar, May–June 2003 to 2015. Table S2 Proportions of confirmed malaria patients (all age groups) among tested patients attending public health care facilities in Micheweni and North A districts between 1999 and 2015. Table S3 All-cause mortality in children < 5 years of age in North A district between 1998 and 2014. Data from Vital Registry. (DOCX 20 kb)

## Abbreviations

ACT: Artemisinin-based combination therapy
AL: Artemether-lumefantrine
AMFm: Affordable medicines for malaria
API: Annual parasite incidence (per 1000 inhabitants)
ASAQ: Artesunate-amodiaquine
HBR: Human biting rate
HRP2: Histidine-rich-protein 2
IRS: Indoor residual spraying
ITN: Insecticide-treated net
LLIN: Long-lasting insecticidal net
MCN: Malaria case notification (system)
OD: Optical density
RDT: Rapid diagnostic test
SCR: Seroconversion rate
WHO: World Health Organization
ZAMEP: Zanzibar Malaria Elimination Programme
