# Supplementary material for: From high to low malaria transmission in Zanzibar—challenges and opportunities to achieve elimination
Source: BMC Med. 2019 Jan 22;17:14. doi: 10.1186/s12916-018-1243-z (PMC6341737; doi:10.1186/s12916-018-1243-z)

**Figure S1**

**Proportions of <5 and ≥ 5 years of age reporting having slept under a bed net the night before and proportion of households reporting having been sprayed the year before the respective surveys between 2003 to 2015 in a) Micheweni district and b) North A district.**


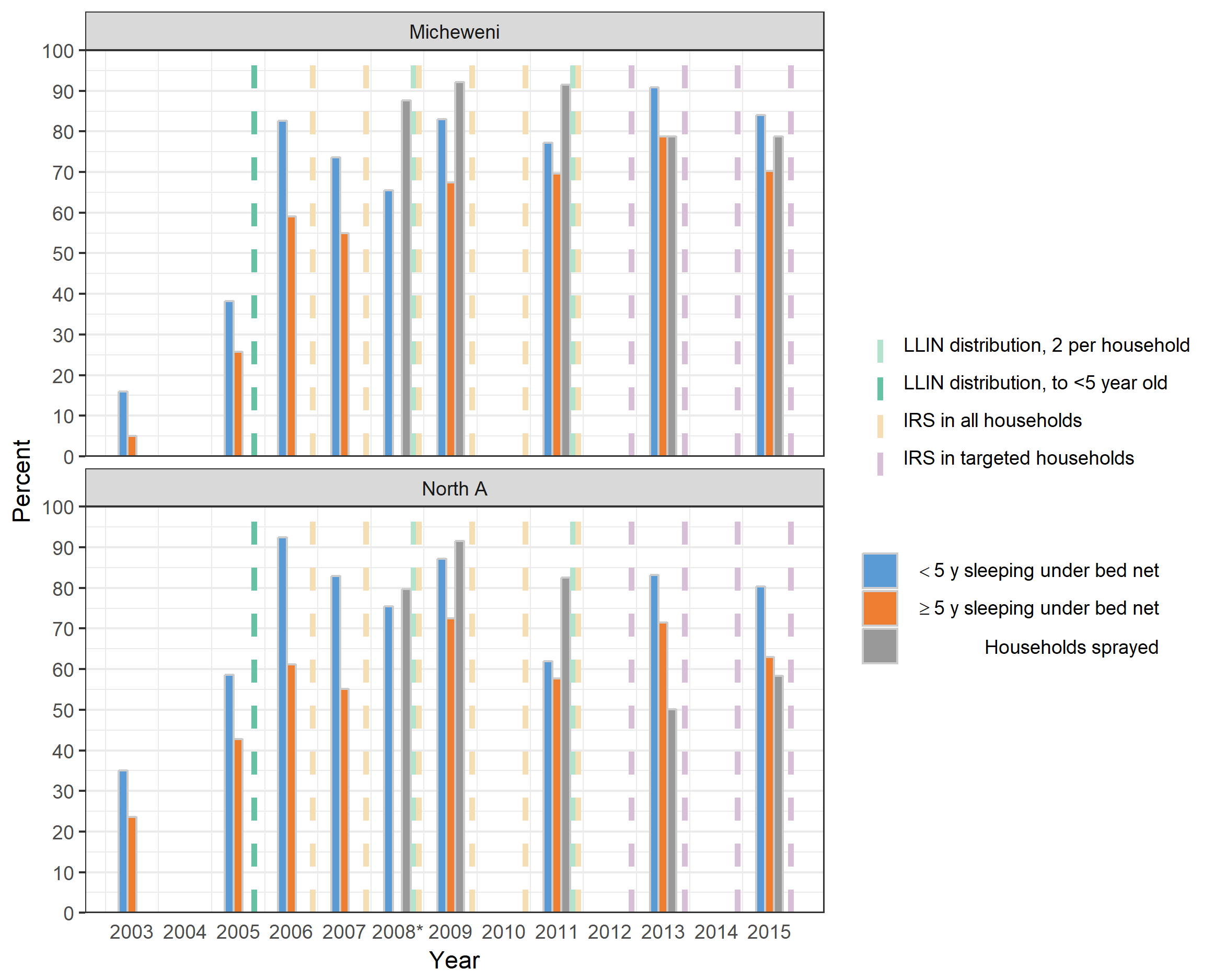


* Data for people >5 years were not available

**Figure S2**

**Proportions of *An. arabiensis* among *An. gambiae* s*.l* mosquito samples collected in surveys on Unguja island, Zanzibar between 2005 and 2014.**


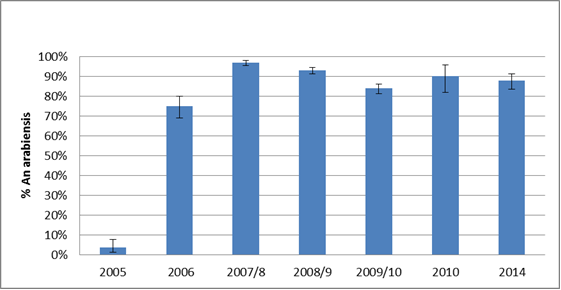

Supplement: Supplementary file 1 — Figure S1. Proportions of < 5 and ≥ 5 years of age reporting having slept under a bed net the night before and proportion of households reporting having been sprayed the year before the respective surveys between 2003 to 2015 in a) Micheweni district and b) North A district. Figure S2 Proportions of An. arabiensis among An. gambiae s.l mosquito samples collected in surveys on Unguja island, Zanzibar between 2005 and 2014. (DOCX 100 kb) [file 12916_2018_1243_MOESM1_ESM.docx]
