# Supplementary material for: From high to low malaria transmission in Zanzibar—challenges and opportunities to achieve elimination
Source: BMC Med. 2019 Jan 22;17:14. doi: 10.1186/s12916-018-1243-z (PMC6341737; doi:10.1186/s12916-018-1243-z)
Supplement: Supplementary file 2 — Table S1. Community prevalences of P. falciparum asexual parasitaemia and gametocytaemia according to microscopy of RDT by age group in North A and Micheweni districts of Zanzibar, May–June 2003 to 2015. Table S2 Proportions of confirmed malaria patients (all age groups) among tested patients attending public health care facilities in Micheweni and North A districts between 1999 and 2015. Table S3 All-cause mortality in children < 5 years of age in North A district between 1998 and 2014. Data from Vital Registry. (DOCX 20 kb) [file 12916_2018_1243_MOESM2_ESM.docx]

**Supplementary tables**

**Table S1.**

**Community prevalences of *P. falciparum* asexual parasitemia and gametocytemia according to microscopy of RDT by age group in North A and Micheweni districts of Zanzibar, May-June 2003 to 2015.**

| *P. falciparum* | Age group | Year | Micheweni | | North A | |
| --- | --- | --- | --- | --- | --- | --- |
|  |  |  | n (%) | (95% CI) | n (%) | (95% CI) |
| Asexual | <5 y | 2003 | 64/298 (21·5%) | 16·8-26·2 | 47/576 (8·2%) | 6·0-10·4 |
|  |  | 2005 | 58/283 (20·5%) | 15·8-25·2 | 15/314 (4·8%) | 2·4-7·2 |
|  |  | 2006 | 26/309 (8·4%) | 5·3-11·5 | 1/315 (0·3%) | 0·0-0·9 |
|  |  | 2007 | 7/395 (1·8%) | 0·5-3·1 | 0/375 (0·0%) | 0·0-0·9 |
|  |  | 2008 | 4/507 (0·8%) | 0·0-1·6 | 1/388 (0·3%) | 0·0-0·8 |
|  |  | 2009 | 0/372 (0·0%) | 0·0-1·0 | 0/281 (0·0%) | 0·0-1·3 |
|  |  | 2011* | 2/265 (0·8%) | 0·0-1·9 | 1/337 (0·3%) | 0·0-0·9 |
|  |  | 2013* | 1/334 (0·3%) | 0·0-0·9 | 0/312 (0·0%) | 0·0-1·2 |
|  |  | 2015 | 2/306 (0·7%) | 0·0-1·6 | 1/330 (0·3%) | 0·0-0·9 |
|  | 5-14 y | 2003 | 76/265 (28·7%) | 23·3-34·2 | 90/657 (13·7%) | 11·1-16·3 |
|  |  | 2005 | 59/323 (18·3%) | 14·1-22·5 | 17/441 (3·9%) | 2·1-5.7 |
|  |  | 2006 | 23/269 (8·6%) | 5·3-12·0 | 6/371 (1·6%) | 0·3-2·9 |
|  |  | 2007 | 6/420 (1·4%) | 0·3-2·5 | 0/387 (0·0%) | 0·0-0·9 |
|  |  | 2008 | 10/637 (1·6%) | 0.6-2.8 | 2/459 (0·4%) | 0·0-1·0 |
|  |  | 2009 | 0/431 (0·0%) | 0·0-0·9 | 0/220 (0·0%) | 0·0-1·7 |
|  |  | 2011* | 3/357 (0·8%) | 0·0-1·7 | 0/398 (0·0%) | 0·0-0·9 |
|  |  | 2013* | 4/483(0·8%) | 0·0-1·6 | 1/389 (0·3%) | 0·0-0·8 |
|  |  | 2015* | 5/488 (1·0%) | 0·1-1·9 | 3/431 (0·7%) | 0·0-1·5 |
|  | >14y | 2003 | 32/626 (5·1%) | 3·4-6·8 | 37/934 (4·0%) | 2·7-5·3 |
|  |  | 2005 | 18/635 (2·8%) | 1·5-4·1 | 16/748 (2·1%) | 1·1-3·1 |
|  |  | 2006 | 7/604 (1·2%) | 0·3-2·1 | 5/747 (0·7%) | 0·1-1·3 |
|  |  | 2007 | 2/760 (0·3%) | 0·0-0·7 | 0/737 (0·0%) | 0·0-0·5 |
|  |  | 2008 | 2/947 (0·2%) | 0·0-0·5 | 1/899 (0·1%) | 0·0-0·3 |
|  |  | 2009 | 0/726 (0·0%) | 0·0-0·5 | 0/662 (0·0%) | 0·0-0·6 |
|  |  | 2011* | 5/649 (0·8%) | 0·1-1·5 | 1/826 (0·1%) | 0·0-0·3 |
|  |  | 2013* | 2/762 (0·3%) | 0·0-0·7 | 2/746 (0·3%) | 0·0-0·7 |
|  |  | 2015* | 2/721 (0·3%) | 0·0-0·7 | 0/736 (0·0%) | 0·0-0·5 |
| Gametocytes | <5 y | 2003 | 3/298 (1·0%) | 0·0-2·1 | 11/576 (1·9%) | 0·8-3·0 |
|  |  | 2005 | 1/283 (0·4%) | 0·0-1·1 | 0/314 (0·0%) | 0·0-1·2 |
|  |  | 2006 | 2/309 (0·3%) | 0·0-0.9 | 3/315(0·9%) | 0·0-1·9 |
|  | 5-14 y | 2003 | 1/265 (0·4%) | 0·0-1.2 | 7/657 (1·1%) | 0·3-1·9 |
|  |  | 2005 | 1/323 (0·3%) | 0·0-0·9 | 3/441 (0·7%) | 0·0-1·5 |
|  |  | 2006 | 0/301 (0·0%) | 0·0-1·2 | 0/371 (0·0%) | 0·0-1·0 |
|  | >14y | 2003 | 2/626 (0·3%) | 0·0-0·7 | 5/934 (0·5%) | 0·1-1·0 |
|  |  | 2005 | 0/635 (0·0%) | 0·0-0·0 | 0/748 (0·0%) | 0·0-0·5 |
|  |  | 2006 | 0/651 (0·0%) | 0·0-0·0 | 2/747 (0·3%) | 0·0-0·7 |

* Parasite detection by RDT

**Table S2**

**Proportions of confirmed malaria patients (all age groups) among tested patients attending public health care facilities in Micheweni and North A districts between 1999 and 2015.**

| **Year** | **Micheweni** | | | **North A** | | |
| --- | --- | --- | --- | --- | --- | --- |
|  | **Tested** | **Confirmed*** | **Positivity rate (95% CI)** | **Tested** | **Confirmed *** | **Positivity rate**  **(95% CI)** |
| 1999 | 10754 | 5506 | 51·2%  (50·2-52·1) | 5181 | 1522 | 29·4%  (28·1-30·6) |
| 2000 | 9221 | 4925 | 53·4%  (52·4-54·4) | 6051 | 1446 | 23·9%  (22·8-25·0) |
| 2001 | 12075 | 5618 | 46·5%  (45·6-47·4) | 5303 | 1843 | 34·8%  (33·4-36·1) |
| 2002 | 11088 | 5125 | 46·2%  (45·2-47·2) | 5919 | 1377 | 23·3%  (22·2-24·4) |
| 2003 | 9881 | 4410 | 44·6%  (43·6-45·6) | 6151 | 1417 | 23·0%  (22·0- 24·1) |
| 2004 | 11972 | 4154 | 34·7%  (33·8-35·6) | 6635 | 1265 | 19·1%  (18·1-20·0) |
| 2005 | 13395 | 2469 | 18·4%  (17·8-19·1) | 8581 | 1059 | 12·3%  (11·6-13·1) |
| 2006 | 16906 | 1255 | 7·4%  (7·0-7·8) | 24990 | 391 | 1·6%  (1·4-1·7) |
| 2007 | 15135 | 955 | 6·3%  (5·9-6·7) | 27937 | 261 | 0·9%  (0·8-1·1) |
| 2008 | 20497 | 354 | 1·7%  (1·6-1·9) | 19020 | 224 | 1·2%  (1·0-1·3) |
| 2009 | 11608 | 305 | 2·6%  (2·3-2·9) | 17512 | 139 | 0·8%  (0·7-0·9) |
| 2010 | 19959 | 336 | 1·7%  (1·5-1·9) | 17185 | 142 | 0·8%  (0·7-1·0) |
| 2011 | 38478 | 292 | 0·8%  (0·7-0·9) | 31799 | 130 | 0·4%  (0·3-0·5) |
| 2012 | 26455 | 654 | 2·5%  (2·3-2·7) | 24606 | 152 | 0·6%  (0·5-0·7) |
| 2013 | 22122 | 255 | 1·2 %  (1·1-1·3) | 17684 | 181 | 1·0%  (0·9-1·2) |
| 2014 | 17719 | 295 | 1·7% (1·5-1·9) | 19793 | 326 | 1·6% (1·4-1·8) |
| 2015 | 19534 | 391 | 2·0%  (1·8-2-2) | 13283 | 316 | 2·4%  (2·1-2·7) |

* Confirmation of malaria diagnosis using microscopy 1999-2005 and microscopy or RDT 2006-2015.

Linear time trends in positivity starting 2012 are decreasing in Micheweni (p < 0.01) and increasing in North A (p < 0.01). Woolf interaction test (p < 0.001).

**Table S3**

**All-cause mortality in children <5years of age in North A district between 1998 and 2014. Data from Vital Registry.**

| **Year** | **Total <5 population** | **Total deaths among infants <1** | **Total deaths among children between 1-4 y** | **Total deaths among children <5 y** | **Total child mortality/year among children <5y (95% CI)** |
| --- | --- | --- | --- | --- | --- |
| **1998** | 11995 | 87 | 74 | 161 | 1·34% (1·14-1·56) |
| **1999** | 12283 | 97 | 68 | 165 | 1·34% (1·15-1·56) |
| **2000** | 12578 | 91 | 66 | 157 | 1·25% (1·06-1·46) |
| **2001** | 12878 | 86 | 45 | 131 | 1·02% (0·85-1·21) |
| **2002** | 13189 | 67 | 66 | 133 | 1·01% (0·84-1·19) |
| **2003** | 13506 | 63 | 62 | 125 | 0·93% (0·77-1·10) |
| **2004** | 13830 | 57 | 40 | 97 | 0·70% (0·57-0·85) |
| **2005** | 14162 | 45 | 19 | 64 | 0·45% (0·35-0·58) |
| **2006** | 14501 | 46 | 18 | 64 | 0·44% (0·34-0·56) |
| **2007** | 14849 | 24 | 11 | 35 | 0·24% (0·16-0·33) |
| **2008** | 15206 | 34 | 15 | 49 | 0·32% (0·24-0·43) |
| **2009*** | 15571 | 20 | 24 | 44 | 0·28% (0·21-0·38) |
| **2010** | 15945 | 35 | 12 | 47 | 0·29% (0·21-0·37) |
| **2011** | 16328 | 71 | 14 | 85 | 0·52% (0·42-0·64) |
| **2012** | 16720 | 19 | 10 | 29 | 0·17% (0·12-0·25) |
| **2013** | 17092 | 37 | 15 | 52 | 0·30% (0·23-0·40) |
| **2014** | 17493 | 50 | 13 | 63 | 0.36% (0·28-0·48) |

*2009 report covered only Jan-June and the figure was doubled as an estimate for the whole year.
